# Nutritional Factors, DNA Methylation, and Risk of Type 2 Diabetes and Obesity: Perspectives and Challenges

**DOI:** 10.3390/ijms20122983

**Published:** 2019-06-19

**Authors:** Luca Parrillo, Rosa Spinelli, Antonella Nicolò, Michele Longo, Paola Mirra, Gregory Alexander Raciti, Claudia Miele, Francesco Beguinot

**Affiliations:** 1 Department of Translation Medicine, Federico II University of Naples, 80131 Naples, Italy; lparrillo@alice.it (L.P.); spinelli.rossella@gmail.com (R.S.); antonellan43@gmail.com (A.N.); mi_longo@libero.it (M.L.); paolamirra06@gmail.com (P.M.); gregoryraciti@gmail.com (G.A.R.); 2 URT Genomic of Diabetes, Institute of Experimental Endocrinology and Oncology, National Research Council, 80131 Naples, Italy

**Keywords:** type 2 diabetes, obesity, DNA methylation, nutritional factors, caloric restriction, high-fat feeding, epigenetic biomarker, personalized medicine, prevention, epigenetic drugs

## Abstract

A healthy diet improves life expectancy and helps to prevent common chronic diseases such as type 2 diabetes (T2D) and obesity. The mechanisms driving these effects are not fully understood, but are likely to involve epigenetics. Epigenetic mechanisms control gene expression, maintaining the DNA sequence, and therefore the full genomic information inherited from our parents, unchanged. An interesting feature of epigenetic changes lies in their dynamic nature and reversibility. Accordingly, they are susceptible to correction through targeted interventions. Here we will review the evidence supporting a role for nutritional factors in mediating metabolic disease risk through DNA methylation changes. Special emphasis will be placed on the potential of using DNA methylation traits as biomarkers to predict risk of obesity and T2D as well as on their response to dietary and pharmacological (epi-drug) interventions.

## 1. Introduction

Obesity and type 2 diabetes (T2D) are responsible for a major reduction in life expectancy and quality of life, increasing healthcare costs. The incidence of obesity and T2D continues to rise and represents a major public health problem. While a genetic component undoubtedly plays a role in determining individual susceptibility to these traits, the genetic loci identified by genome-wide association studies (GWASs) only explain a small fraction of the heritability of these disorders (less than 2% for obesity and 5–10% for T2D) [1,2]. Some studies have led to the conclusion that genetic factors only marginally contribute to obesity and T2D development and transmission within families. Indeed, known polymorphisms have little impact on current strategies to predict obesity and T2D, even when multiple genes are simultaneously taken into account through risk algorithms. Thus, genetic studies have so far left the issue of how metabolic disorders are transmitted unsolved. These low heritability estimates, along with the dramatic increase in their worldwide prevalence, suggest a major role of lifestyle factors in these diseases [3]. 

Epigenetics is the study of inheritable and reversible phenomena that modulate gene function without affecting the genome sequence [4]. It represents a logical interface between the genome and environment which shapes T2D and obesity risk and may help explaining the “missing heritability” [5]. 

The two main epigenetic mechanisms underlying metabolic disorders are DNA methylation and histone modifications. Histone modifications include a series of complex post-translational changes such as methylation (mono-, di-, and tri-methylation), acetylation, and SUMOylation. Post-translational changes in histone proteins can alter the chromatin conformation and gene regulation, contributing thus to the metabolic disease phenotype. Their influence on the development of obesity and T2D has been well-described elsewhere [6], and will not be covered in the present review, which is specifically focused on DNA methylation. 

The current epidemic of obesity and T2D is mainly due to the current unhealthy lifestyle and nutrition [7]. As one of the primary contributors, dietary imbalances, either undernutrition or overnutrition (both during the prenatal or postnatal life), lead to epigenetic reprogramming which is associated to increased T2D and obesity incidence [8]. For instance, it has been shown that high-fat feeding induces distinctive changes in the mouse epigenome that can be transgenerationally transmitted [9,10]. This treatment also alters the plasticity of DNA methylation in low birth weight human subjects [11]. Interestingly, suboptimal caloric intake during the early stages of development (pregnancy or lactation) can disturb the correct allocation of epigenetic marks, increasing the risk of obesity and T2D in the offspring [12]. In this regard, an unbalanced maternal diet may also impact the early establishment of the fetal and neonatal microbiome, leading to specific epigenetic signatures that may potentially predispose to the development of late-life obesity and T2D, as described in [13]. Consistently, adequate nutrition enables proper DNA methylation of obesity and T2D genes and reduces disease risk [14]. In mice, 5-month treatment with a high-fat diet (HFD) intake caused hypermethylation of the *Hoxa5* gene promoter in visceral fat. HFD replacement with a standard chow diet is accompanied by simultaneous improvement of the metabolic phenotype and rescue of normal methylation levels at *Hoxa5* [15]. All together, these findings have uncovered the capacity of epigenetics to adaptively respond to different nutritional conditions and suggest that targeting epigenetic plasticity may, in the future, offer novel and realistic opportunities to prevent and treat obesity and T2D. In addition, novel evidence now indicates that, in humans, epigenetic changes may represent potential biomarkers predicting individual lifetime risk of obesity and T2D before the development of the disease phenotype [16]. 

In this review, we will focus on DNA methylation and its potential role in mediating the risk of metabolic disorders caused by unhealthy nutrition. The potential use of DNA methylation marks in the evaluation of disease risk and efficacy of intervention will also be discussed.

## 2. DNA Methylation

DNA methylation is the most widely studied epigenetic mark and is well conserved among most plant, animal, and fungal models [17]. In mammals, it is based on covalent addition of methyl groups to DNA bases. Three conserved enzymes, DNA methyltransferase 1 (*DNMT1*), *DNMT3A*, and *DNMT3B*, are responsible for its occurrence and maintenance and are essential for normal development. Among these, *DNMT1* is responsible for the maintenance of a methylation profile that will be conserved through cellular duplication, whereas *DNMT3A* and *DNMT3B* are de novo methyltransferases and are important for DNA methylation at the early embryonic stage [18].

DNA methylation marks mainly occur at the 5′ position of the cytosine residues of cytidine-guanine dinucleotides (CpG, where p indicates the phosphate group between the two nucleotides). Clustered CpG dinucleotides forming dense repeat sequences in the genome are termed CpG islands. Islands are located especially in promoter regions. However, they can also be found in intragenic and enhancer regions [19]. Many studies have revealed that DNA methylation also occurs at sites other than the CpG sequences (non-CpG methylation). Non-CpG methylation has been suggested to be prevalent in human embryonic stem cells and in the brain. However, its functional significance in the mammal genome is poorly understood and will not be further discussed here [20].

DNA methylation is associated with either gene repression or activation depending on the location where it occurs. Generally, DNA methylation in regions near the transcription start site and in the enhancer regions suppresses gene expression [21]. The transcriptional suppression by DNA methylation involves inhibition of transcriptional activation factor binding or recruitment of transcription inhibiting factors to these regions [22]. Gene body methylation rather correlates to gene expression and may prevent a spurious transcript initiation from DNA repetitive elements or alternative promoters [23].

Cytosine methylation (5mC) has long been considered a stable epigenetic modification capable of surviving meiosis. However, 5mC can be converted back to cytosine by either passive or active demethylation [24]. Passive demethylation can be caused by the inhibition of *DNMT1* during cell replication [25], while the active DNA demethylation is catalyzed by DNA demethylases termed ten-eleven translocation (TET) proteins. These enzymes convert 5-methylcytosine to 5-hydroxymethylcytosine and promote a locus-specific reversal of the DNA methylation [26,27].

There is broad evidence indicating that DNA methylation is essential for mammalian development and plays an important role in gene silencing, in protection against spurious repetitive element activity, in genomic stability during mitosis, and in parent-of-origin imprinting [28]. Modulation of DNA methylation is crucial in many biological processes. Hematopoiesis represents a benchmark model for the role of DNA methylation in adult stem cells and in later lineage specification. Indeed, all myeloid and lymphoid blood lineages are differentiated from hematopoietic stem cells [29], which are achieved through the activity of a number of genes controlling cell fate tightly regulated by their DNA methylation status [30]. Conversely, aberrant DNA methylation profiles contribute to the occurrence of several human diseases, including cancer, autoimmune diseases, neurological defects, and metabolic disorders [31,32].

Methyl groups for DNA methylation originate from the universal methyl donor S-adenosyl-methionine (SAM) [33]. SAM is synthesized in the methionine cycle by several precursors present in diet, including methionine, folate, choline, betaine and the B2, B6, and B12 vitamins. All of them enter the methionine cycle at different sites and contribute to SAM synthesis [34]. Deficiencies in these nutrients may result in changes in the SAM pool, which can also influence DNMT activity and DNA methylation. The establishment and maintenance of the Methylome is therefore vulnerable to nutritional factors. Indeed, nutritional challenges throughout a person’s lifetime might have a major impact on DNA methylation, as well on development and the individual’s health.

## 3. Time Points of Plasticity in the Epigenetic System

It is now well-established that different dietary regimens can alter epigenetic processes and may therefore contribute to increasing the susceptibility to metabolic disorders including obesity and T2D. A further question is at which time points during ontogenesis and subsequent life the epigenetic system is more sensitive to nutritional factors, which may ultimately have long-term effects on gene expression profiles. 

Broad evidence suggests that, in mammals, early development (especially fetal development and/or early neonatal growth) is the most critical period for establishment of the genome-wide epigenetic profiles. Single mismatches at this time may result in disadvantageous changes causing embryonic lethality, developmental malformations, and an increased risk of disease, including metabolic disturbances [13]. At this critical stage in development, exceptionally rapid cell differentiation and complex epigenetic remodelling occur, making ontogenesis particularly vulnerable to nutritional insults, that can disrupt the correct make up of epigenetic marks that, once established, remain highly stable [35]. Hence, early development is a period of exceptional epigenome plasticity, and represents a time when nutritionally-induced epigenetic errors may have major consequences for the health.

However, it is also becoming clearer that nutritional effects on the epigenome may occur throughout the entire life time, especially during long-term dietary transitions [36]. Dietary transitions are prolonged periods of time (ranging from weeks to months in animal models to years in humans) during which individuals are exposed to diets characterized by malnutrition (both under- and over-nutrition). These situations may cause subtle and long-lasting changes in gene expression. Some of these changes are mediated by epigenetic mechanisms that, while potentially reversible, remain stable and may seriously affect phenotypes. 

**Figure 1:**
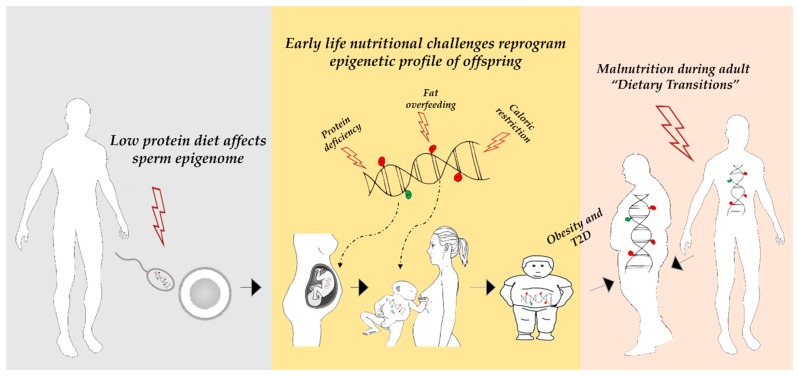
Altered nutritional exposures during the life time can induce persistent changes in DNA methylation, leading to an increased susceptibility to obesity and type 2 diabetes (T2D). Left: paternal low protein diet alters spermatozoa methylome. This may programme the epigenetics of the germ line which, in turn, may be inherited and influence offspring metabolic disturbance risk. Middle: early development is a period particularly vulnerable to nutritional challenges that can disrupt the correct make up of DNA methylation marks that, once established, remain highly stable. Right: exposure to a prolonged period of diet characterized by malnutrition throughout the life, may cause permanent epigenetically-associated changes in gene expression that can contribute to shape the disease phenotype.

Hence, nutritional exposures occurring during all stages of the life time can have persistent consequences on health or disease risk. In this review, we will focus on and discuss intervention studies examining the impact of different diets on mammal DNA methylation and shaping the metabolic disease phenotype. The reported interventions include protein deficiency, fat overfeeding and caloric restriction (CR) (Figure 1). The first section is dedicated to the Agouti mouse model which serves as a key example of the association between diet and DNA methylation.

## 4. The Agouti Mouse Model 

Perhaps the most striking example of how DNA methylation changes induced by maternal diet during pregnancy can dramatically alter the phenotype in the offspring is offered by the agouti viable yellow (*Avy*) mouse [37]. The agouti-related neuropeptide (*Agrp*) gene encodes a paracrine factor that stimulates the production of a yellow pigment (pheomelanin) rather than the black pigment (eumelanin) in the follicles of the hair. The transcription of the gene from a wild-type allele is restricted to the hair follicles and results in the characteristic brown color of the wild-type mice. The *Avy* allele results from the transposition of a murine retrotransposon (intracisternal A particle, IAP) upstream of the agouti gene. The agouti gene expression in the other cells is regulated by the methylation of this IAP locus, that acts as a cryptic promoter. An aberrant expression of the agouti gene gives rise to the yellow fur, obesity, diabetes and tumorigenesis (the agouti disease phenotype). 

Interestingly, CpG methylation in the *Avy* IAP inversely correlates with ectopic agouti expression. Thus, the isogenic offspring vary in agouti expression depending on the availability of the developmental methyl group. If a pregnant agouti mouse receives a dietary supplement that can release the methyl groups—such as folic acid, vitamin B12, or choline—the pups’ agouti genes become methylated and thus inactive. These pups still carry the agouti gene but they lose the agouti disease phenotype: they have brown fur and no increased tendency to develop metabolic disorders and cancer. In addition, these effects are also inherited in the F2 generation suggesting a germline transmission of the epigenetic changes [38]. Thus, suboptimal early nutrition alters the establishment of the DNA methylation profiles in the fetus and has adverse metabolic effects on the offspring’s health, clearly showing the direct link between diet, DNA methylation, and the increased risk of obesity and diabetes in adulthood.

## 5. Nutritional Factors Affecting DNA Methylation

### 5.1. Protein Malnutrition 

A low-protein diet, frequently used as a model for maternal malnutrition, has been reported to induce changes in DNA methylation and metabolic disturbances that may persist into adulthood.

In rodent models, it has been shown that a maternal protein deficiency during pregnancy results in global DNA hypermethylation in the offspring livers. This methylation pattern has been associated with alterations in glucose tolerance and hypertension in adults [39]. A suboptimal protein intake during pregnancy may also induce locus-specific alterations in DNA methylation. These changes in DNA methylation correlated with altered gene expression (or function), and, importantly, remained stable until adulthood. The affected genes play a significant role in key metabolic processes, including liver lipid metabolism (glucocorticoid receptor (*GR*) [40]; peroxisome proliferator-activated receptor alpha (*PPARα*) [41]; liver X receptor alpha (*Lxrα*) [42]), pancreatic β-cell function (hepatocyte nuclear-4-alpha (*Hnf4α*) [43]), and appetite and energy balance regulation (orexigenic/anorexigenic genes neuropeptide Y and proopiomelanocortin C (*Nyp*) [44]; leptin (*Lep*) [45]). Taken together, these findings indicate that a maternal low protein diet persistently alters DNA methylation profiles and function of several genes important in metabolism. It is possible that some of these nutritionally-induced epigenetic alterations may explain, at least in part, the development of obesity, insulin resistance, and T2D in adults (Figure 1). 

In addition, a paternal low protein diet also seems to induce changes of DNA methylation. Watkins et al. [46] have recently developed a mouse paternal low-protein diet (LPD) model to determine LPD impact on semen quality, maternal uterine physiology, and adult offspring health. They have demonstrated that paternal LPD induces sperm-DNA hypomethylation in conjunction with blunted female reproductive tract embryotrophic, immunological, and vascular remodeling responses. Interestingly, they relate the sperm- and seminal plasma-specific epigenetic programming effects of the paternal diet with an elevated offspring adiposity, metabolic dysfunction, and altered gut microbiota. Thus, nutritional deficiencies in paternal diet may also programme the epigenome of the germ line which, in turn, may be inherited and influence offspring metabolic disease risk (Figure 1).

### 5.2. Global Caloric Restriction 

#### 5.2.1. Animal Models 

A moderate restriction of energy intake during early development is associated with epigenetic modifications that persist in the adult [47]. A maternal energy restriction alters DNA methylation in the insulin-like growth factor 2/H19 imprinted maternally expressed transcript (*IGF2/H19*) gene. An aberrant DNA methylation at *IGF2/H19* locus at birth increases the risk of excessive weight and obesity in children [48]. In addition, CR in pregnant baboons has been shown to increase the expression of the gluconeogenic enzyme *PCK1* in the fetal liver, along with *PCK1* promoter hypomethylation [49]. These alterations were suggested to have negative effects on fetal metabolism and cause metabolic dysfunction later in life.

#### 5.2.2. Human Studies

In humans, the first well-documented evidence of nutrition impact on the individual epigenetic profile comes from investigation on women who were prenatally exposed to famine during the Dutch Hunger Winter. In an elegant study, Heijmans and colleagues [50] demonstrated that the insulin-like growth factor 2 (*IGF2*) locus was less methylated in the white blood cells of individuals who had been prenatally exposed to famine when compared to matched controls. Subsequent investigations have described altered DNA methylation in other genes in the famine exposed-group, including *INSIGF2* (INS-IGF2 Readthrough), *GNASAS1* (GNAS antisense RNA 1)*, MEG3* (Maternally expressed 3), *IL-10* (Interleukin-10), and *LEP*, some of which have a known role in metabolic disorders [51]. These findings have initially substantiated the concept that a differential DNA methylation at these genes links early nutrition to adult metabolic disease. Interestingly, the association reported in these studies was specific for periconceptional exposure, reinforcing the hypothesis that early-life environmental conditions can cause epigenetic changes in humans that persist throughout the adult life (Figure 1).

Caloric restriction has attracted considerable attention due to its effects on health. It has been shown to be capable of extending the lifespan and delaying the onset of chronic diseases, including obesity and T2D [52] (Figure 1). Caloric restriction may exert its beneficial effects through epigenetic mechanisms as well. Short-term calorie restriction on obese subjects may revert the aberrant DNA methylation at specific loci, including Wilms tumor 1 (WT1) and Tumor necrosis factor-alpha (*TNF-α*) [53,54]. In adult humans, Bouchard et al. [55] reported a different methylation profile and transcriptomic difference in the adipose tissue in high compared to low responders to caloric restriction. The genes identified in these studies were associated to body weight control and insulin secretion. Fasting periods can also influence health and disease through DNA methylation. For example, individuals with low birth weight (LBW) have an increased risk of insulin resistance and T2D in adult life, and they respond differently to prolonged fasting than individuals with normal birth weight (NBW). The differential response to fasting was associated with increased DNA methylation at *PPARGC1A* in the muscle of subjects with a LBW [56]. More recently, Line Hjort et al. [57] analysed the effects of a 36-hour fast on DNA methylation and expression of the adiponectin (*ADIPOQ*) and leptin (*LEP*) genes in the subcutaneous fat from the same subjects previously studied by the authors of [56]. The *LEP* and *ADIPOQ* DNA methylation levels increased with 36-hour fasting in NBW subjects only. In contrast, the *ADIPOQ* mRNA levels increased in LBW males, whereas the *LEP* expression did not change with fasting. This differential response to fasting led the authors to propose that LBW subjects may be more inflexible in altering their DNA methylation status when metabolically challenged, and therefore are at greater risk of metabolic disturbance.

### 5.3. High-Fat Feeding

High-fat feeding may also induce DNA methylation changes at key metabolic genes that could influence their expression and predisposing to metabolic dysfunctions [58]. 

#### 5.3.1. Animal Models 

In the context of early life nutrition, maternal high-fat feeding during pregnancy affects DNA methylation and the expression of genes related to dopamine and opioid systems in the brain of the offspring [59]. This seems to increase behavioral preferences for palatable foods, thereby contributing to the risk of a metabolic disorders. 

The lactation-suckling period in mice causes epigenetic changes that influence risk of developing obesity later in life. Milk lipids have been shown to activate the *PPARα* gene [60], a key regulator of the hepatic liver metabolism. By using a whole-genome DNA methylation analysis, Yuan et al. [61] identified few *PPARα* target genes, including fibroblast growth factor-21 (*Fgf21*), that undergo ligand-activated *PPARα*-dependent DNA demethylation during the perinatal period. *Fgf21* is an endocrine factor that regulates glucose uptake, metabolism, and energy expenditure [62]. Interestingly, once established in early life, the DNA methylation levels at *Fgf21* remain stable in adulthood. Low levels of *Fgf21* methylation correlates with protection from diet-induced obesity in older animals. These same authors further demonstrated that in newborn mice lactation keeps *Fgf21* methylation to low levels. In a further recent study, Butruille et al. [63] showed that maternal high-fat feeding during lactation induced qualitative changes in breast milk fatty acid (FA) composition (high n-6/n-3 polyunsaturated FA ratio and low medium-chain FA content). As a consequence, the offspring of adults fed HFD were predisposed to weight gain and showed increased visceral adipose tissue growth. Remarkably, these events were associated with an altered DNA methylation profile of the stearoyl-CoA desaturase-1 (*SCD1*), a key enzyme of the fatty acid metabolism. These findings suggest a link between breastfeeding and the susceptibility for weight gain and adiposity, and elegantly describe how specific DNA methylation marks established early in life have long-lasting effects (Figure 1). 

In adult rodents, HFD has been reported to induce DNA methylation changes in the white adipose tissue (WAT) at loci close to genes controlling metabolism [64]. Our own studies have shown that 5-month HFD feeding alters promoter methylation and expression of the *Ankrd26* gene [65]. In mice, this gene is highly expressed in both the hypothalamus and WAT and its partial inactivation induces marked hyperphagia, severe obesity and diabetes *in vivo*, while in humans *ANKRD26* has been associated to certain forms of hereditary obesity [4]. More recently we have also shown that *ANKRD26* mRNA levels are negatively correlated with body mass index (BMI) in humans. In addition, we have further uncovered that HFD treatment produced adipose tissue abnormalities accompanied by epigenetic changes at the *Hoxa5* adipose tissue remodeling gene [15]. The *Hoxa5* gene was highly methylated at its 5’UTR and transcriptionally repressed in the WAT from HFD-induced obese mice. Interestingly, when obese mice exposed to the chronic HFD intervention were returned to standard chow diet (STD) for a further two-month period, the *Hoxa5* DNA methylation and expression levels returned to values similar to those of mice maintained under a STD since the inception of the study. These findings not only emphasize the dynamic nature of DNA methylation but also strongly suggests that *Hoxa5* may represent a potential tool to quantify the obesity response to nutritional intervention. The most relevant findings of the above-mentioned studies are reported in Table 1. 

#### 5.3.2. Human Studies

Only a few studies have been carried out assessing the relationship between overfeeding and changes in DNA methylation in humans (Table 2). A five-day overfeeding intervention resulted in alterations of both gene expression and methylation patterns in human adipose tissue and skeletal muscle [11,66,67]. Interestingly, these changes were not fully reversed by low-caloric diet, even after 6–8 weeks, suggesting that changes in methylation at certain loci may accumulate over time. In another study, *PPARGC1A* promoter methylation in the skeletal muscle increased after five days of overfeeding [68]. In this case, however, the methylation levels were reversed after the introduction of the low caloric diet.

Recent findings have suggested that not only the amount but also the composition of dietary fat may have profound effects on the risk of contracting a metabolic disease such as obesity, insulin resistance or T2D [69]. A randomized control study examined the impact of a 7-week diet of either excessive saturated or polyunsaturated fats on the global DNA methylation pattern in human adipose tissue. Both diets resulted in a similar weight gain and increased methylation in the adipose tissue. However, the DNA methylation of individual genes and CpG sites was differently regulated by the saturated and polyunsaturated fatty acid administration. Genes that were involved in the carbohydrate metabolism, lipid metabolism, and oxidative phosphorylation were among the most significantly affected by these interventions (Table 3) [70]. Finally, *in vitro* exposure to palmitate of human pancreatic islets resulted in global DNA hyper-methylation along with changes in the expression of several genes which may contribute to impaired insulin secretion observed in these studies [71].

## 6. DNA Methylation and Future Medicine

As described in this review, DNA methylation changes in key metabolic tissues may contribute to the pathogenesis of obesity and T2D. This has attracted interest in the identification of epigenetic biomarkers that, in the future, may prove effective in the prediction of metabolic disorders. To achieve this objective, most studies concentrated on blood cells because of their accessibility and diagnostic accuracy [72]. Importantly, obesity- and T2D-associated DNA methylation changes in metabolically active tissues (pancreatic β-cells, adipose tissue, liver and muscle) have been reported to be quite often reflected in the blood-borne cells, suggesting that blood samples might be used as a surrogate of major metabolic organs [72]. The prognostic significance of similar DNA-based epigenetic biomarkers has already been demonstrated in cancer patients [73].

A number of studies have investigated the potential of using DNA methylation markers to identify subjects at increased risk and to predict the incidence of metabolic diseases in different prospective cohorts [74]. Godfrey et al. [75] reported that epigenetic analysis at the perinatal stage may be used to predict adiposity occurring in childhood. In particular, the methylation of the *RXRA* (Retinoid X receptor alpha) promoter at specific sites in the umbilical cord tissue DNA correlated with the level of adiposity at six and nine years of age. *RXRA* controls the adipose tissue metabolism and insulin sensitivity. Similarly, a low DNA methylation level at the FTO (FTO alpha-ketoglutarate dependent dioxygenase) gene represents an early marker of T2D. FTO is a well-known risk gene for obesity and T2D [76]. Its effect on the disease phenotype has been recently reported to be functionally connected with the regulation of the *IRX3* (Iroquois homeobox 3) gene, an important determinant of body mass and composition [77]. Importantly, the predictive power of *RXRA* and *FTO* methylation are significantly greater than that of any genetic variants so far described. This further suggests that epigenetic analysis at the perinatal stage may identify the risk of metabolic alterations with unprecedented power and accuracy. More recently, other studies have shown a suggestive link between DNA methylation and adiposity. Sharp et al. [78] reported that the offspring of obese mothers displayed a number of CpG sites differently methylated in the cord blood when compared with the offspring from lean mothers. In this regard, epigenetic dysregulation of several genes (*Nna*t and *Peg3*) of the *Trim28*-imprinted gene network also influences the risk for the offspring of developing obesity, as elegantly described by Dalgaard et al. [79]. Additionally, the DNA methylation signature in blood cells is distinct between early-onset obese and control individuals [80]. Moreover, Dick et al. [81] described an association between an increased BMI and an increased methylation at the *HIF3A* locus in blood cells and in adipose tissue of the same subjects, suggesting that DNA methylation perturbation of hypoxia-inducible factor (HIF) signaling might represent a mark of increased body adiposity.

Several studies have shown that disturbances in DNA methylation may predict future development of T2D. Chambers et al. [82] analyzed T2D-associated DNA methylation changes in blood samples from 2664 Indian Asians and replicated the study findings in 1141 Europeans. These authors uncovered that altered methylation at the *ABCG1* (ATP binding cassette subfamily G member 1), *PHOSPHO1* (Phosphoethanolamine/phosphocholine phosphatase), *SOCS3* (Suppressor of cytokine signaling 3), *SREBF1* (Sterol regulatory element binding transcription factor 1), and *TXNIP* (Thioredoxin interacting protein) loci was strongly associated with future development of T2D. Remarkably, the combined methylation scores of these loci predicts future risk of T2D, independently of traditional T2D risk factors, including family history of T2D, BMI, physical activity, and hemoglobin A1c (HbA1c). These methylation marks were further analyzed in the Botnia prospective cohort where only the association between *ABCG1* and *PHOSPHO1* methylation and future T2D risk was confirmed [83]. These authors found that methylation at the *ABCG1* locus associated with increased risk, whereas methylation at *PHOSPHO1* associated with a decreased risk of future T2D. These changes could be replicated in the blood samples of diabetic twins compared to their non-diabetic counterparts [83]. The association between *ABCG1* locus methylation and T2D incidence has recently been reported in independent studies [84,85]. *ABCG1* plays a major role in cholesterol and phospholipid transport [86]. Cholesterol abnormalities are hallmark of T2D. This may suggest that an altered DNA methylation at the *ABCG1* locus may also influence circulating cholesterol levels, and thus have an impact on the onset and progression of T2D and other metabolic disturbances. Aging is associated with an increased T2D risk. In this context, Bacos et al. [87] demonstrated that blood-borne epigenetic marks reflect age-related DNA methylation changes in human islets, and that altered methylation at certain genes are associated with the future insulin-secreting capacity and future risk of T2D. Finally, in an epigenome-wide association study (EWAS), Wahl et al. [88] identified, in whole blood samples from ~10,000 individuals, 187 CpG sites significantly associated with obesity. Interestingly, DNA methylation changes at these sites predicted the future risk of T2D.

DNA methylation may be used as a marker to predict the response to treatment as well. Successful intervention in diseased individuals state are indeed expected to change methylation levels. For instance, in the intervention study published by Cordero et al. [89], 27 obese women were prescribed an 8-week low-calorie diet. At the end of the dietary treatment, 21 subjects presented a positive response to the diet (>5% weight loss) whereas the others were considered non-responders (<5% weight loss). Interestingly, the responders had lower DNA methylation levels in the promoter of the *LEP* and *TNF-alpha* genes than non-responders at baseline. These findings suggest that methylation levels at these loci could be used to predict the susceptibility to weight loss by nutritional intervention. In addition, those subjects who successfully maintained weight loss for three years from inception of diet have DNA methylation levels that are similar to normal-weight individuals but different from their obese counterparts [90]. A nutritional educational weight loss program in overweight and obese adolescents also revealed that the methylation status of genes related to glucose metabolism, insulin resistance, and inflammation was associated to weight control and glucose as well as lipid metabolism [91]. A physical activity program further identified strong associations between the methylation profile at a number of CpG sites and weight loss in blood samples from 20 healthy women [92]. Finally, Perfilyev et al. [70] found a significant association between DNA methylation in adipose tissue at baseline and weight increase in response to excess energy intake. 

As previously mentioned, a further promising aspect of epigenetic changes lies in their dynamic nature and potential reversibility under targeted interventions. This ambitious task might be accomplished by normalizing/optimizing modifiable and lifestyle-sensitive epigenetic factors (e.g., nutritional factors, physical activity) or by using epigenetic modulating agents. Several epigenetic modifier agents have so far been identified, including DNMT inhibitors (e.g., azacytidine) and histone deacetylase inhibitors (HDACi) (e.g., valproic acid (VPA) and trichostatin A (TSA)). Medications targeting epigenetic mechanisms have been used in oncology for a long time [93]. Epigenetic drugs may also have beneficial effects on T2D and obesity risk. There is mounting evidence showing that HDACs regulate glucose homeostasis and islet function [94]. For instance, MC1568, an HDACi, improves insulin secretion in pancreatic islets from human donors with T2D [95]. MC1568 inhibits HDAC7, the overexpression of which in clonal ß cells and rat islets results in impaired glucose-stimulated insulin secretion and mitochondrial dysfunction [96]. One of the epi-drugs tested for T2D is valproic acid, but the results from these studies have been inconclusive thus far [97,98]. Recently, Bridgeman et al. [99] reviewed the epigenetic effects of metformin. This well-known antidiabetic agent modulates the activity of numerous epigenetic modifying enzymes, such as HDACi, histone acetyltransferases (HATs), and DNMTs, influencing thus the epigenome and gene expression. It has been argued that these effects may contribute to the antidiabetic properties of metformin. Despite a significant degree of uncertainty existing in relation to the overall effect of metformin on the epigenome, this medication holds great promise in targeting the epigenome to treat T2D and other metabolic disturbances. 

There are also concerns related to the use of epigenetic drugs in metabolic dysfunction. First, the low specificity and global action of these agents may cause adverse effects. Secondly, the number of epigenetic changes that must be reverted is uncertain. In cancer, the DNA methylation of larger genomic areas is robustly modified, and the differences between normal and malignant cells are more evident, so that epigenetic drugs can be used in relatively high doses [100]. In metabolic diseases, the differences in DNA methylation between affected and non-affected individuals are more modest (about 10–20% at most), and mainly locus/i-specific [101]. Therefore, the development of molecules/substances with a more selective modulatory action on epigenetic processes should represent a real challenge to develop therapeutic strategies in the future. The search for nutrients and compounds found in food that are able to slightly modify the epigenome in key organs related to obesity and T2D holds great promises for obtaining functional foods that can help in preventing or combating metabolic diseases.

## 7. Conclusions

The onset of obesity and T2D seems to be due to the interplay between post-natal and fetal environment and inherited genetic factors that determine the individual’s susceptibility to developing the disease phenotype. However, growing evidence suggests that one of the links between the environmental factors and the higher predisposition to developing obesity and T2D is DNA methylation. The studies outlined above establish that, to a significant extent, nutritional factors act by modulating DNA methylation, and that some of these factors might be used in obesity and T2D therapy due, at least in part, to their modulatory action on epigenetic mechanisms. Importantly, nutritional challenges from the prenatal stage to adulthood can lead to alterations in DNA methylation that influence the risk of developing obesity and T2D. 

The blood-cell DNA methylation signatures in T2D and obesity have been tested and hold promise as surrogate markers for metabolic diseases. These findings will have important implications for the development of non-invasive tests, given the difficulty in accessing key tissues for obesity and T2D, particularly for longitudinal studies. Profiling DNA methylation in blood could be used to monitor high-risk individuals, delay or prevent the onset of T2D and obesity, and facilitate early intervention strategies. Most importantly, the dynamic and reversible nature of epigenetic mechanisms offers unique opportunities for the development of strategies for the treatment and prevention of obesity and T2D. Nonetheless, the pathway towards translating basic research into clinical application is still challenging.

## Figures and Tables

**Table 1:** List of relevant nutrition-sensitive DNA methylation loci in humans and model organisms.

Dietary Condition	DNA Methylation Regulated Locus(i)	Sample Type(s)	Species	References
Low protein diet	*GR, PPARα, Hnf4a, Nyp, Pomc*	Liver, pancreatic islets, hypothalamus	Rat	[40,41,43,44]
	*Lxrα, Lep*	Liver, adipose tissue	Mouse	[42,45]
Caloric restriction	*IGF2/H19*	Adrenal gland	Sheep	[47]
	*PCK1*	Fetal liver	Baboon	[49]
	*IGF2, INSIGF2, GNASAS1, MEG3, IL-10, LEP*	Blood	Human	[50,51]
	*WT1, TNF-α*	Blood	Human	[53,54]
	*PPARGC1A*	Skeletal muscle	Human	[56]
	*ADIPOQ, LEP*	Adipose tissue	Human	[57]
High-fat feeding	*Fgf21*	Liver	Mouse	[61]
	*Scd1*	Adipose tissue	Rat	[63]
	*Ankrd26, Hoxa5*	Adipose tissue	Mouse	[15,65]

**Table 2:** Effects of a 5-day high-fat overfeeding diet on DNA methylation in humans.

Study Population	Sample Type	Methylation Strategy	Epigenetically Regulated Genes	Reference
Healthy men (*n* = 21)	Skeletal muscle	Genome-wide *	*DNM2*, *MGMT*, *SLC2A3/GLUT3*, *MRC1*, *ACAT2*, *APOH*, *DCC*, *ESRRG*, *FOLH1*, *GTF2I*, *MC4R*, *MYST4*, *AKT2*, *PDX1/IPF1*, *SLC30A8*, *CDKN2A*, *CDKN2B*, *PPARG*	[67]
NBW (*n* = 23), LBW (*n* = 17)	Skeletal muscle	Genome-wide *	*IGF2R*, *TNF*, *CDKN2B*, *KCNJ11*, *KCNQ1*, *GABRA3*, *UGT2B7*, *FOLH1*, *FUT1*, *NDUFS2*, *FAP*	[11]
NBW (*n* = 24), LBW (*n* = 16)	Subcutaneous adipose tissue	Genome-wide *	*ACAT1*, *CPLX1*, *FADS2*, *GPRC5B*, *HCCA2*, *IGF2R*, *CIDEA*, *KLF14*, *PRDM16*	[66]
NBW (*n* = 26), LBW (*n* = 20)	Skeletal muscle	Gene-specific	*PPARGC1A*	[68]

NBW, normal birth weight; LBW, low birth weight. * Regarding the genome-wide studies, only the top scored DNA methylated genes are shown.

**Table 3:** Impact of 7 weeks of overfeeding with SFAs or PUFAs on DNA methylation in humans.

Study Population	Sample Type	Methylation Strategy	Epigenetically Regulated Genes	Reference
LIPOGAIN cohort (*n* = 31; SFA group *n* = 17; PUFA group *n* = 14)	Subcutaneous adipose tissue	Genome-wide *	*RPSAP9*, *ADIPOQ*, *FABP1*, *FABP2*, *FABP7*, *MC2R*, *XKR4*, *MC3R*, *MC5R*, *PPARGC1A*, *TNF*, *ACO1*, *SLC37A2*, *AR*, *CXCL2*, *FOXO1*, *FTO*, *IL6*, *INSR*, *MC1R*, *MC3R*, *MEF2A*, *NEGR1*, *POMC*	[70]

SFAs, saturated fatty acids; PUFA, polyunsaturated fatty acids. * Top scored affected genes are presented.

## Abbreviations

T2D	Type 2 diabetes
GWAS	Genome-wide association studies
HFD	High-fat diet
DNMT	DNA methyltransferase
C*p*G	Cytidine-phosphate-Guanine
5mC	5-methyl cytosine
TET	Ten-eleven translocation
SAM	S.-adenosyl-methionine
CR	Caloric restriction
Avy	Agouti viable yellow
Agrp	Agouti related neuropeptide
IAP	Intracisternal A particle
GR	Glucocorticoid receptor
PPARα	Peroxisome proliferator-activated receptor alpha
Lxrα	Liver X receptor alpha
Hnf4α	Hepatocyte nuclear factor 4 alpha
Nyp	Neuropeptide Y
Pomc	Pro-opiomelanocortin
LEP	Leptin
LPD	Low-protein diet
IGF2/H19	Insulin like growth factor 2/ H19 imprinted maternally expressed transcript
PCK1	phosphoenolpyruvate carboxykinase 1
INSIGF2	INS-IGF2 Readthrough
GNASAS1	GNAS antisense RNA 1
MEG3	Maternally expressed 3
IL-10	Interleukin-10
WT1	Wilms tumor 1
TNF-α	Tumor necrosis factor alpha
LBW	Low birth weight
NBW	Normal birth weight
PPARGC1A	Peroxisome proliferative activated receptor, gamma, coactivator 1 alpha
ADIPOQ	Adiponectin
Fgf21	Fibroblast growth factor 21
FA	Fatty acid
SCD1	Stearoyl-coenzyme A desaturase 1
WAT	White adipose tissue
ANKRD26	Ankyrin repeat domain 26
Hoxa5	Homeobox A5
5’UTR	5’ untranslated region
STD	Standard diet
RXRA	Retinoid X receptor alpha
FTO	FTO alpha-ketoglutarate dependent dioxygenase
IRX3	Iroquois homeobox 3
HIF3A	Hypoxia inducible factor 3 subunit alpha
Nnat	Neuronatin
Peg3	Paternally expressed 3
Trim-28	Tripartite motif-containing 28
ABCG1	ATP binding cassette subfamily G member 1
PHOSPHO1	Phosphoethanolamine/phosphocholine phosphatase
SOCS3	Suppressor of cytokine signaling 3
SREBF1	Sterol regulatory element binding transcription factor 1
TXNIP	Thioredoxin interacting protein
BMI	Body mass index
HbA1c	Hemoglobin A1c
EWAS	Epigenome-wide association study
HDACi	Histone deacetylase inhibitors
VPA	Valproic acid
TSA	Trichostatin A
HAT	Histone acetyltransferases
